# Microdialysis as a tool for antibiotic assessment in patients with diabetic foot: a review

**DOI:** 10.3389/fendo.2023.1141086

**Published:** 2023-04-17

**Authors:** Vladimíra Fejfarová, Radka Jarošíková, Jan Polák, Blanka Sommerová, Jitka Husáková, Veronika Wosková, Michal Dubský, Petr Tůma

**Affiliations:** ^1^ Diabetes Centre, Institute for Clinical and Experimental Medicine, Prague, Czechia; ^2^ Second Faculty of Medicine, Charles University, Prague, Czechia; ^3^ Department of Pathophysiology, Third Faculty of Medicine, Charles University, Prague, Czechia; ^4^ Department of Hygiene, Third Faculty of Medicine, Charles University, Prague, Czechia

**Keywords:** antibiotic (ATB), capillary electrophoresis (CE), diabetic foot (DF), diabetic foot infection (DFI), microdialysis (MD)

## Abstract

Diabetic foot is a serious late complication frequently caused by infection and ischaemia. Both require prompt and aggressive treatment to avoid lower limb amputation. The effectiveness of peripheral arterial disease therapy can be easily verified using triplex ultrasound, ankle-brachial/toe-brachial index examination, or transcutaneous oxygen pressure. However, the success of infection treatment is difficult to establish in patients with diabetic foot. Intravenous systemic antibiotics are recommended for the treatment of infectious complications in patients with moderate or serious stages of infection. Antibiotic therapy should be initiated promptly and aggressively to achieve sufficient serum and peripheral antibiotic concentrations. Antibiotic serum levels are easily evaluated by pharmacokinetic assessment. However, antibiotic concentrations in peripheral tissues, especially in diabetic foot, are not routinely detectable. This review describes microdialysis techniques that have shown promise in determining antibiotic levels in the surroundings of diabetic foot lesions.

## 1 Background

The connection between diabetic foot (DF) and microdialysis seems to be clinically irrelevant, since in daily clinical practice microdialysis is not routinely used, only obviously for research purposes. With a smaller number of published studies to date, the aim of this review is to provide further insights on this neglected issue.

## 2 Diabetic foot

DF is defined as infection, ulceration, or destruction of tissues of the foot in a patient with currently or previously diagnosed diabetes mellitus. The condition is usually accompanied by neuropathy and/or peripheral arterial disease (PAD) in the lower extremities (1). DF therapy requires a comprehensive diagnostic and therapeutic approach, focusing on different factors that may contribute to the development of this late diabetes complication. Management of DF typically involves local treatment and detection of biomechanical abnormalities resolved by appropriate off-loading methods and, in certain cases (diagnosed PAD), endovascular or surgical revascularisation. However, attention should also be paid to the type of infection and its complications.

### 2.1 Diabetic foot infection

Diabetic foot infection (DFI) is clinically defined by signs and symptoms of redness, heat, swelling, and pain. Nearly half of all diabetic foot ulcers are classified as infected (2, 3). If DFI is left untreated, local infection can quickly develop to widespread overt infection involving other tissues such as bone (3). Thereafter, osteitis or osteomyelitis develops. Severe DFIs have negative impacts on health-related quality of life, and can result in limb-threatening and life-threatening scenarios (4). Minor amputations are necessary in approximatelly 40% of patients and major amputations in approx. 20% of patients with DFI (5). Significant risk factors for lower extremity amputation include severe infections, osteomyelitis, and certain bacterial strains.

Various diabetic foot risk classification systems have been developed to predict foot complications, clinical management (6, 7), and mainly prognosis. Monteiro-Soares et al. reviewed the available systems used to classify diabetic foot ulcers in order to synthesise methodological and qualitative issues and to determine their accuracy in predicting lower extremity amputation. Across 25 studies, the authors reported a prevalence of lower extremity amputation of between 6% and 78%. The Meggitt-Wagner, S(AD) (size (area and depth) SAD (sepsis, arteriopathy, denervation), and University of Texas classification systems were the most extensively validated, other 10 classification systems derived or validated only once (8). The Wound, Ischemia, and foot Infection (WIfI) classification system is used to precisely establish DF prognosis, factoring in wound depth, severity of ischaemia, and foot infection (9). More recently, the Laboratory Risk Indicator for Necrotizing Fasciitis (LRINEC) score has shown promise in predicting both amputation and mortality in DFI (10).

To prevent lower limb amputation as well as patient morbidity and mortality, infectious DF complications must be treated promptly and aggresively. The type of anti-infectious therapy depends on the severity of DFI, patient comorbidities, and the availability of comprehensive care. Local uncomplicated infections are usually treated using modern local devices with antibacterial effect. More advanced DFIs should be preferably treated using systematically administered antibiotics (ATB). Locally applied ATBs are not routinely used, since they can theoretically contribute to, or induce, bacterial ATB resistence (11). On the other hand, local ATB carriers can be administered to the target region to induce very high ATB concentrations, resulting in maximal bactericidal action in the goal peripheral tissue. A study by Fletcher et al. demonstrated that the local use of calcium sulphate beads containing a combination of two ATBs (vancomycin + gentamicin or flucloxacillin + rifampicin) demonstrated high efficacy against polymicrobial DFI flora and individual bacterial strains using an *in vitro* zone of inhibition assay (even in the case of *Staphylococcus aureus* and *Pseudomonas* spp.) (12).

In cases where DFI is treated by ATBs, the recommended duration of therapy is 1-2 weeks for mild forms of infection, 2-4 weeks for moderate cases, and 4 weeks or longer for severe forms of DFI (12). Osteomyelitis is a specific podiatric problem typically resolved using long-term ATB therapy lasting 6-8 weeks or longer in the case of conservative management (12). Recently, however, there is a trend to reduce ATB treatment in the case of osteomyelitis, with a recommended maximum duration of 4-6 weeks or even shorter in selected cases (13).

Previously, the literature sources on ATB treatment efficacy in patients with DFI were scarce, since tissue concentrations of selected ATBs in peripheral tissue or liquid samples were detectable only once. From these analyses, it was not possible to make a comprehensive overview of how effectively patients with diabetic foot are treated, especially in the presence of PAD or other modifing factors. Therefore, a novel methods how to get continuously peripheral tissue samples or better liquid were discovered and introduced. Many techniques for obtaining wound fluid have been described. There is very little validation data, and the array of different techniques appears confusing. Structuring and new standards are needed to avoid inaccuracies in wound fluid sampling. Most of the wound fluid parameters analysed to date have yet to be introduced into clinical practice (14).

## 3 Microdialysis

Microdialysis is a minimally invasive sampling technique designed primarily for *in vivo* monitoring of metabolic, biochemical, physiological, and pharmacological processes in living tissues and organs (15). Originally, microdialysis was developed for rapid monitoring of neurotransmitter levels in the central nervous system (CNS) (16, 17). Today, it is widely (18) used to monitor metabolite dynamics in most organs and tissues (19). Biologically active substances are mainly determined from blood plasma, serum, and urine. Less traditional samples include cerebrospinal fluid, saliva, sweat, expectoracy condensate, etc. Recently, also an extracelullar liquid from different parts of the body (adipose tissue, soft tissue, muscles, bones) have also been sampled (19). In addition, microdialysis is widely applied in pharmaceutical research, where it is used to assess drug pharmacokinetics and pharmacodynamics in peripheral tissues through non-invasive transdermal sampling (18, 20, 21).

### 3.1 Factors influencing concentrations of substances in microdialysate

Microdialysis is a continuous sampling technique for in-vivo monitoring of small water-soluble substances in the extracellular environment of tissues and organs. As it is a low-invasive sampling technique with minimal impact on biogenic processes, it is widely used to monitor the pharmacokinetics of drugs in the subcutaneous tissue (22). The microdialysis device consists of a microdialysis probe equipped with a semipermeable hollow membrane, which is connected via an inlet tube to a syringe pump with saline solution and to an outlet tube for collecting microdialysate. The probe is implemented in the subcutaneous tissue and flushed with perfusate solution at a constant flow rate of 0.5 - 5.0 μL/min (15). The perfusate rinses the membrane from the inside and during the flow through the probe is enriched with metabolites that are transported from the tissue across the membrane into the perfusate based on their concentration gradient. Only substances whose molecular weight is less than the porosity of the membrane material, expressed as a cut-off, pass through the membrane. To achieve a high microdialysis recovery, expressed as the ratio between the concentration of the substance in the microdialysate versus the concentration in the tissue, it is necessary to use a cut-off that is 4 times higher compared to molecular weight (23). Saline (Ringer) solution is used as a perfusion solution, which simulates the composition of extracellular fluid, thus minimizing undesired transport of substances across the membrane in and out due to differences in osmolarity (16).

### 3.2 Practical application of microdialysis

**Figure 1:**
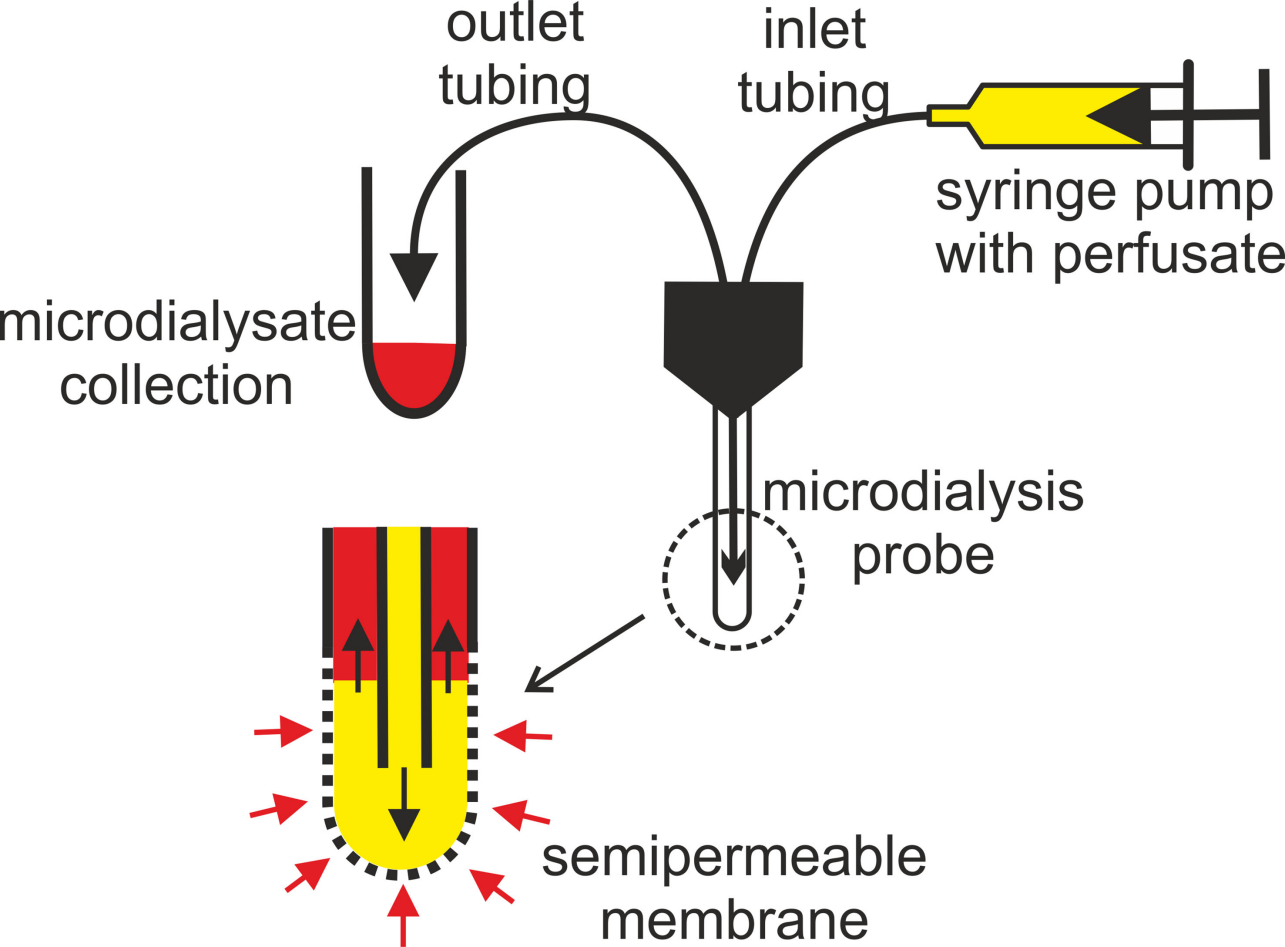
Principle of microdialysis tissue sampling showing a probe tip equipped with a selective membrane. Black arrows indicate the direction of perfusate flow; red arrows indicate the direction of metabolite diffusion; perfusate (yellow), microdialysate (red).

Several microdialysis probes have been designed, including a linear probe for peripheral tissues, a rigid pin-style probe for brain microdialysis, a flexible probe for intravenous use (all commercially supplied by CMA Microdialysis; http://microdialysis.com/), and a shunt probe for bile duct sampling. The most common design is a flexible concentric probe primarily used in soft-tissue microdialysis (see **Figure 1**). The perfusate of choice is physiological or Ringer’s solution, which blocks the massive transport of major inorganic cations and anions across the membrane, allowing metabolites and drugs to be retrieved for MD analysis.

At the discretion of the investigator, microdialysis probes can be placed in any tissue or organ in the body (24), including the liver (25), heart (26), muscle (27), and skin (28, 29). These probes act in a manner similar to a blood vessel, allowing substances to be removed or delivered to a specific site through diffusion with no net fluid loss. Fibrosis and other tissue reactions can restrict long-lasting microdialysis analysis assessment (several days). However, in the case of DFI, microdialysis normally lasts from 8 to a maximum of 24 hours (30–32). Acute complications in connection with short-lasting microdialysis implantation include inflammation, haemorrhage, and oedema (33). However, they occur seldom and rarely have a negative influence on probe recovery. The microdialysis probe can also be used in reverse mode. In this instance, the drug is added to the perfusion solution, which then diffuses from the probe into the surrounding tissue. This process is called retrodialysis, principally used for the local administration of drugs into the bloodstream (15, 19).

The resulting MD sample most often consists of an aqueous saline solution enriched in low-molecular-weight analytes and devoid of macromolecular substances. Moreover, the volume of MD obtained in this way can vary from few microliters to several tens of microlitres. For these reasons, it is necessary to combine microdialysis with microanalytical techniques (19, 34–37). The most common are highly selective biosensors or mass spectrometry methods combined with direct sample injection or high-performance liquid chromatography (HPLC), most often with a capillary column. Separation techniques such as capillary or microchip electrophoresis performed in capillaries and channels with very small internal diameters represent other important tools in MD analysis (38–40).

## 4 Capillary electrophoresis and microdialysis

Capillary electrophoresis (CE) is an efficient separation technique widely used to determine substances in highly complex matrices obtained as clinical samples, including MD (41, 42). CE is performed in fused silica capillaries with inner diameters of 10-75 μm and lengths of 30-100 cm. Separation is driven by an electric field of up to 1 kV/cm. Since the volume of sample injected into the capillary is in the nanoliter range, microlitre quantities of MD obtained are considered sufficient. In addition, the separation time of CE is significantly shorter than that of HPLC, making CE a suitable technique for rapid sequential monitoring of biological processes. For all these reasons, CE in combination with highly sensitive laser-induced fluorescence (LIF) (43) has become a fundamental technique for monitoring neurotransmitters in the MD of CNS (35, 44).

CE analysis of MD is normally performed in off-line mode, where MD samples are collected at the clinical site, frozen, and then transported to the laboratory. The thawed samples are first laboratory-processed using standard procedures such as filtration, dilution, preconcentration, derivatisation, etc. and subsequently determined on a CE instrument. A more modern approach offers on-line interfacing of MD with CE (38, 45), most commonly implemented using a cross flow gating interface (FGI) (46–50). Cross-FGI is a microfluidic junction with four inputs/outputs for connecting: (a) the inlet to the separation capillary for CE; (b) the output of the tubing that supplies the microdialysate from the MD probe; (c) the tubing outlet supplying the gating solution from the injection pump, which also serves as the background electrolyte (BGE) for CE separation, this flow is repeatedly interrupted by a 3-port valve; (d) the inlet to the stainless steel tube draining the solution to waste, which is also the grounding electrode, the high voltage electrode is located in the output vial with BGE (48). In the basic mode, FGI operates by deflecting the continuous delivery of microdialysate away from the inlet to the separation capillary by BGE flow, which has an order of magnitude higher flow rate. In this mode, the high voltage is switched on and CE analysis is performed. In the injection mode, the BGE delivery to the FGI is stopped by switching the three-way valve, the sample accumulates in the injection compartment and is injected into the inlet of the separation capillary by applying a vacuum to the output vial. Subsequently, the BGE supply is restored, which flushes the excess sample from the injection compartment to the waste, and separation is initiated by switching on the high-voltage power supply. The capillary is washed with BGE between analyses by pressurizing the end vial. This sequence of steps is repeated and the FGI operates in sequential mode (50). This interface enables sequential analysis of MD at intervals ranging from a few tens of seconds to several minutes, which means that the controlling factor for sequencing is the CE separation time. The introduction of the on-line mode eliminates the manual processing of the MD sample, which is associated with its loss. The result is a further reduction in the volume of MD for CE determination, which may be as low as 1 μL.

Microchip electrophoresis is an innovative analytical approach performed in thin channels formed on a glass, quartz, or plastic plate only a few cm^2^ in size (51–53). In a system of interconnected channels, individual steps of the analysis (sample preparation, derivatisation, electrophoretic separation, detection, etc.) are continuously linked to create an entirely miniaturised and automated analytic process.

**Figure 2:**
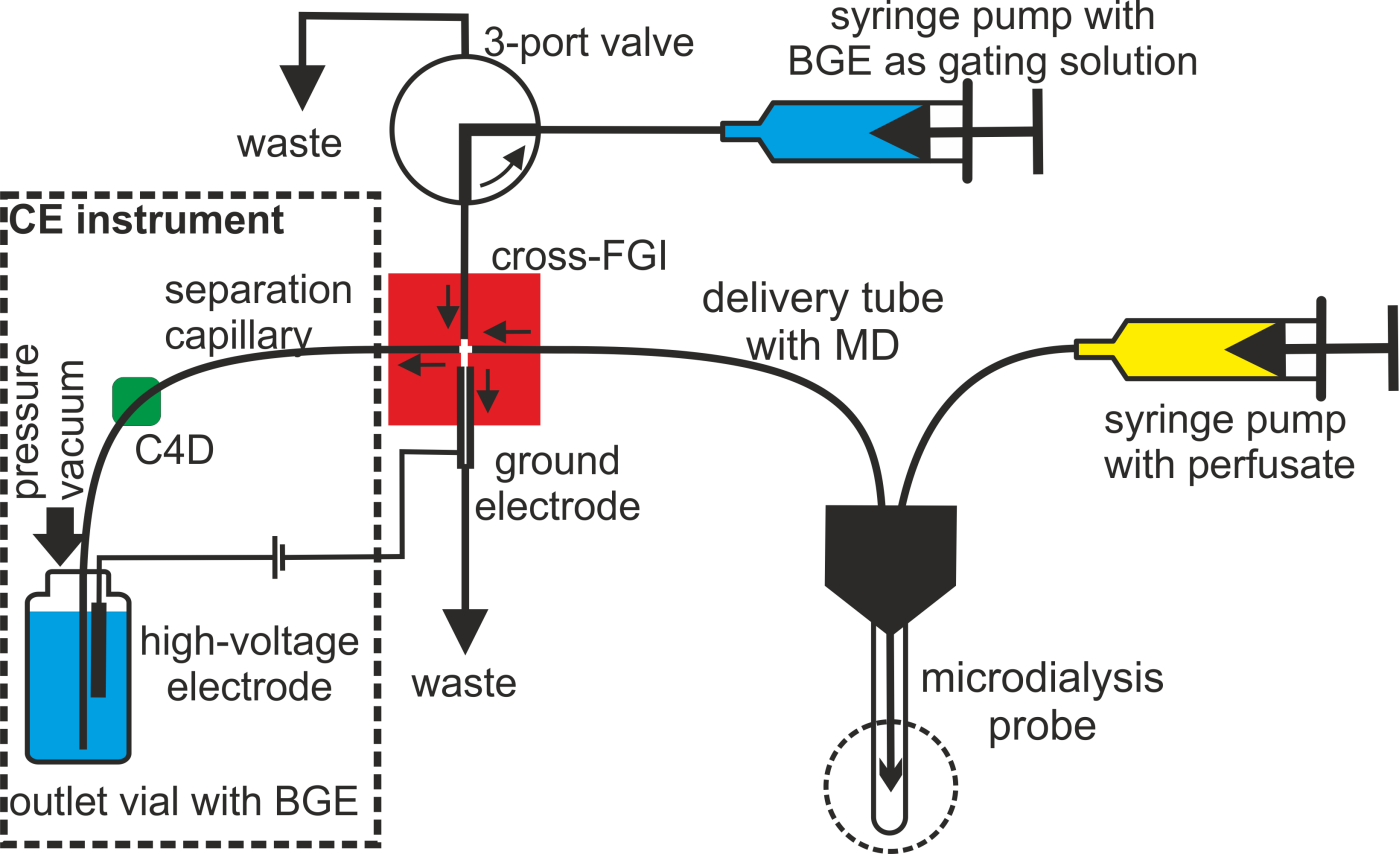
Schematic of the on-line connection of MD sampling with sequential CE analysis implemented using a cross - FGI (red). A detailed description of the FGI function is presented in the text.

**Figure 3:**
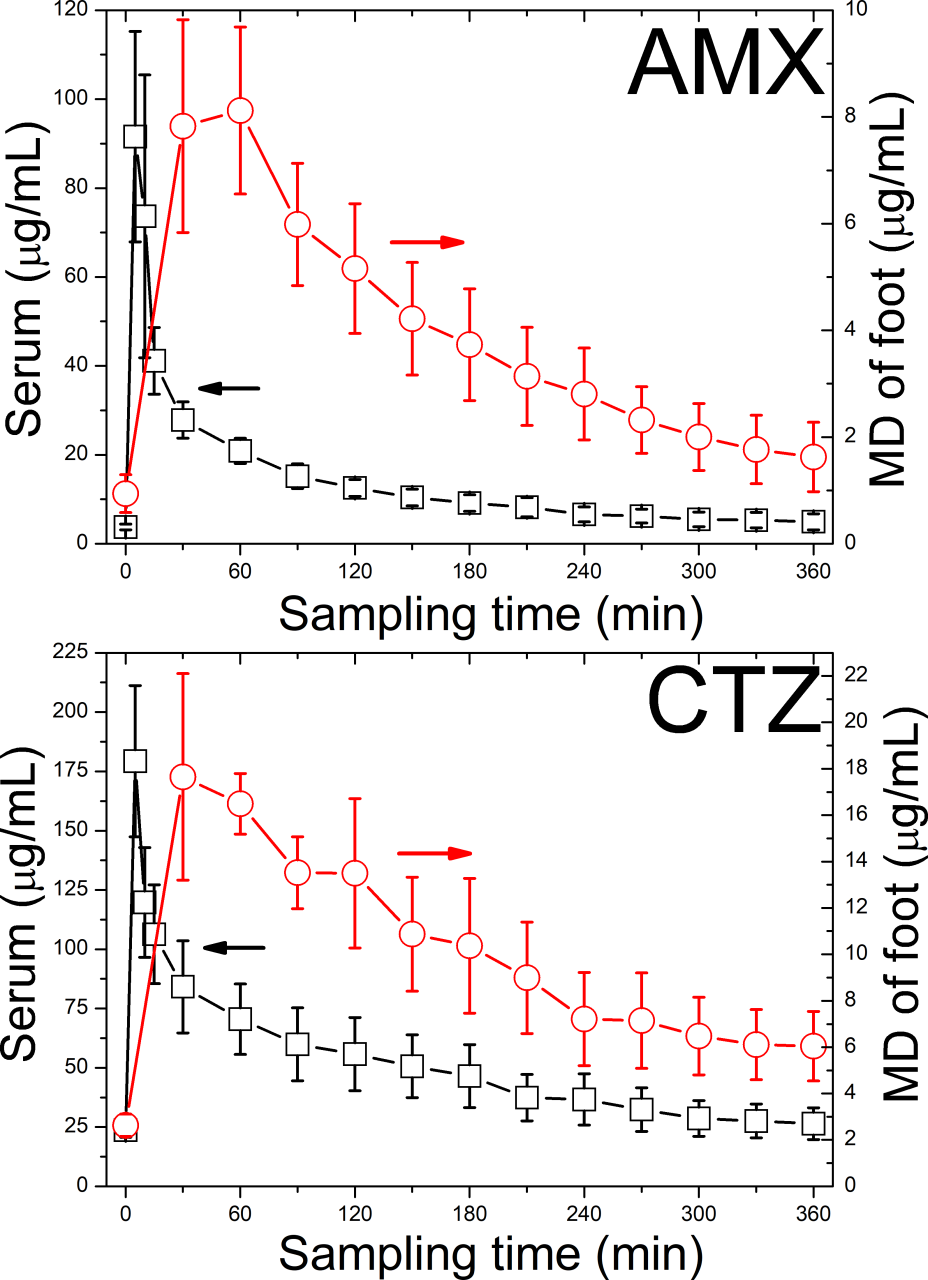
AMX and CTZ in the serum and MD of subcutaneous tissue in DF patients following i.v. bolus administration of AMOKSIKLAV (containing 1.2 g AMX) in 5 patients and FORTUM (containing 2.0 g CTZ) in 3 patients. The error bars indicate the standard error of mean (SEM). The determination was performed by the CE-C4D method with application of large volume sample stacking, which is described in the text and details in the original paper (60).

An alternative MD approach is to combine CE with capacitively coupled contactless conductivity detection (C4D) (54–56). C4D is a universal detection technique that is particularly sensitive for low-molecular-weight substances that dissociate into ions, such as minerals, amino acids, carbohydrates, amines, organic acids, as well as many pharmaceuticals including ATBs (57, 58). Using CE-C4D, metabolites and drugs are determined in their native forms commonly found in living tissues, without the need for the complicated derivatisation associated with CE-LIF (59). This greatly simplifies the analysis of small MD volumes, where the only treatment required is to dilute the MD with an organic solvent before proceeding to direct CE-C4D analysis. CE-C4D detection limits typically range from micromolar to submicromolar concentration levels, which are adequate for most metabolic and pharmacological applications (**Figure 2**). For example, the pharmacokinetics of the ATBs amoxicillin (AMX) and ceftazidime (CTZ) in the blood serum and MD of DF patients can be sequentially monitored after i.v. administration of a single dose of ATB. The collected MD and serum samples are mixed with acetonitrile and the treated sample is determined off-line for both ATBs by the CE-C4D method as previously described (60), details in **Figure 3**.

## 5 Diabetic foot and microdialysis

In a recent review focussed on microdialysis in the context of DF (33), Ray and colleagues found that many antimicrobial agents provide adequate *in vitro* activity against pathogens frequently implicated in DFIs. Although many antimicrobial agents display good *in vitro* activity against the pathogens frequently implicated in DFIs, effective treatment can be complicated by reduced tissue penetration in this population secondary to PAD and emerging antimicrobial resistance. The authors also emphasise the need for better characterisation of ATB tissue pharmacokinetics and penetration ratios in DFI (33).

Microdialysis could be a method of choice for the evaluation of ATB efficacy in the therapy of DFI. The majority of studies published on this topic have been performed using linear probes in off-line mode, with tissue fluid collected in tubes for subsequent analysis. The results of these experiments are typically determined by the microdialysis flow rate, recovery of analytes, and sensitivity of the analytical method used (15, 19).

With regard to the use of microdialysis in DFI patients treated with ATBs, several parameters are useful when assessing ATB efficacy and concentrations in peripheral tissue. These pharmacokinetic/pharmacodynamic (PK/PD) indices include maximum ATB concentration (*C*_max_), biological half-life (*t*_1/2_), the area under the concentration-time curve (AUC), the AUC_tissue_ (where AUC_tissue_ is the AUC from time zero to the time of the last measureable concentration in tissue for certain ATB/AUC_plasma_ for certain ATB) (61), MIC = 100 (area under the concentration-time curve/minimum inhibitory concentration (MIC), and fT (the percentage of a 24-hour period where the unbound drug concentration exceeds the MIC) (62).

A number of microdialysis studies focusing on patients with DFIs have been performed. A study by Gill et al. assessed the plasma and soft-tissue pharmacokinetic exposure of omadacycline (30). A member of the aminomethylcycline subclass of tetracycline ATBs, omadacycline is a broad-spectrum ATB medication approved for the treatment of community-acquired bacterial pneumonia as well as acute bacterial skin and skin structure infections (ABSSSI). *In vitro* studies have shown that omadacycline counteracts a broad range of Gram-positive and select Gram-negative pathogens, including methicillin-resistant *Staphylococcus aureus* (MRSA) and vancomycin-resistant *Enterococcus*. In terms of design, the Gill study was notable for it use of microdialysis catheters *in situ* for 24 hours. A comparative study by Stainton et al. presented pharmacokinetic and tissue penetration data for oral tedizolid in hospitalised patients with DFIs and healthy volunteers (31). Belonging to the second generation of oxazolidinone-class ATBs, tedizolid is an agent marketed for the treatment of ABSSSIs. Compared to linezolid, tedizolid displays greater antimicrobial potential (4-to-16-fold) against staphylococci and enterococci. Participants in the Stainton study received 200 mg of oral tedizolid phosphate every 24 h for a total of 3 doses to achieve a steady state. A microdialysis catheter was inserted into subcutaneous tissue near the margin of the wound in the case of patients, or into the thigh tissue in the case of volunteers. Following the third dose, samples were collected over 24 hours. Like the Gill study, despite lower plasma concentrations and a delay in the time taken to reach peak concentration (*t*_max_) in patients with DFI, penetration into tissue were similar in both DFI patients and healthy volunteers.

Ceftolozane-tazobactam is another promising antibiotic agent with potent activity against DFI, especially infections caused by Gram-negative bacteria. In a study by Monogue et al., the pharmacokinetics and tissue penetration of ceftolozane-tazobactam were evaluated using *in vivo* microdialysis in 10 diabetic subjects and 6 healthy volunteers (61). The authors have confirmed that in the case of *Pseudomonas aeruginosa*, ceftolozane-tazobactam is the most potent (highest AUC in peripheral tissue) agent. A study by Minichmayr et al. described the pharmacokinetics of linezolid also in DFIs *via* serum and microdialysis assessment performed in subcutaneous adipose and muscle tissue. The authors highlighted the impact of covariates on the attainment of pharmacokinetic/pharmacodynamic targets (AUC/MIC = 100 and fT > MIC = 99%) using pooling analysis. The highest clearance of linezolid was detected in septic patients. Penetration into subcutaneous adipose tissue was lowest in diabetic patients (-34.9%) compared with healthy volunteers. Renal functions and body weight were found to influence linezolid exposure. After standard linezolid doses, patients with sepsis and conserved renal functions were at high risk of insufficient ATB peripheral tissue concentrations (62). A study by Eslam et al. assessed tissue concentrations of linezolid in the infected and non-infected tissue of 10 patients suffering from type 2 diabetes and DFI. Tissue penetration of linezolid was assessed using *in vivo* microdialysis at the site of infection as well as in non-inflamed subcutaneous adipose tissue. In contrast to the Minichmayr study, penetration of linezolid was not impaired in DFI compared to non-infected tissue. However, the final results may have been modified by either a different type of tissue in which ATB concentrations were measured or the target tissue blood supply, which is impaired in subcutaneous adipose tissue (63). A similar study was performed by Wiskirchen et al. (64). Another small case study by Traunmuller et al. studied linezolid penetration into the inflamed soft tissue and bone of diabetic patients suffering from severe bacterial foot infections. Despite being of methodological interest, only 3 patients were enrolled in the study. Linezolid concentrations were recorded for plasma, the healthy subcutis, the inflamed subcutis, and cancellous bone (65).

Hamada et al. evaluated the penetration of vancomycin into interstitial tissue fluid from the bloodstream using a microdialysis technique, again involving a limited number of patients. Based on a three-compartment model simulation, the probability of target attainment (PTA) values for 1 g of vancomycin every 12 h and every 8 h in tissue fluid were 39.6% and 56.6%, respectively, at an MIC of 1 mg/L. The mean and median penetration ratios into tissue of the simulated population were 1.91 and 0.85, respectively. The low vancomycin concentration in tissue was probably due to the wide variability in penetration in peripheral tissue. Serum concentration proved an unreliable predictor of exposure in diabetic patients (66). ATB penetration may be enhanced by other approaches based on adjunctive treatment, such as hyperbaric oxygen therapy. In their microdialysis study, Koomanachai et al. demonstrated that hyperbaric oxygenation increases peripheral ATB concentrations almost two-fold (67).

A study by Sauermann et al. involving a small number of patients determined whether ertapenem concentrations at the target site would be sufficient for bacterial killing in patients with DFI. At a steady state, ertapenem concentrations were measured over 8 h in plasma and in the interstitium of healthy subcutaneous adipose tissue and soft tissue adjacent to the foot infection using microdialysis. And although total plasma concentrations of ertapenem in diabetics were lower than values documented in healthy subjects, tissue concentrations were in diabetics similar to those known from healthy volunteers. Moreover irrespective to PAD, ATB concentrations in infected tissue were higher in diabetics compared to healthy subcutaneous adipose tissue (32).

**Table 1** shows the summary of studies with microdialysis techniques used for the ATB concentration assessment.

**Table 1:** A summary of peripheral tissue sampling using microdialysis techniques to detect ATB levels in the diabetic foot.

ATBs	Cut-off (kDa)/MD probe	Method	C(max) – tissue (μg/mL)	C(max) – plasma (μg/mL)	Ref.
*Ciprofloxacin*	*20/CMA 10*	*HPLC*	*2.12 – 2.18*	*2.83*	(68)
*Fosfomycin*	*20/CMA 10*	*GC*	*136 – 139*	*320*	(69)
*Piperacillin*	*20/CMA 10*	*HPLC/UV*	*91.7 – 102.2*	*341*	(70)
*Tazobactam*	*20/CMA 10*	*HPLC/UV*	*5.2 – 10.4*	*15.8*	(70)
*Daptomycin*	*20/CMA 60*	*HPLC/MS*	*3.8 – 4.3*	*62.4 – 67.8*	(21)
*Fosfomycin*	*20/CMA*	*HPLC/UV*	*185.1*	*377.3*	(71)
*Daptomycin*	*20/CMA*	*HPLC/UV*	*4.0 – 4.1*	*72.9*	(72)
*Linezolid*	*20/CMA*	*HPLC/UV*	*13.9 – 17.4*	*22.4*	(65)
*Tigecycline*	*20/CMA 60*	*HPLC*	*0.16 – 0.18*	*0.42*	(73)
*Linezolid*	*20/CMA 60*	*HPLC*	5 – 6*	9 – 10*	(67)
*Linezolid*	*20/CMA 60*	*HPLC*	*13.45 - 14.4*	*11.99*	(64)
*Ertapenem*	*20/CMA*	*HPLC*	*2.4 – 4.5*	*59.4*	(32)
*Linezolid*	*20/CMA 63*	*HPLC*	*6.6 – 6.7*	*16.4*	(63)
*Linezolid*	*20/CMA 60*	*HPLC*	4*	14*	(62)
*Ceftolozane*	*20/CMA 63*	*HPLC*	*33.8 – 39.2*	*55.2 – 91.5*	(61)
*Tazobactam*	*20/CMA 63*	*HPLC*	*6.3 – 7.1*	*14.2 – 17.5*	(61)
*Tedizolid*	*20/CMA 63*	*HPLC*	0.8 – 1.1**	*1.5 – 2.7*	(31)
*Omadacycline*	*20/CMA 63*	*HPLC/MS*	0.04 – 0.1*	0.5 – 1.1*	(30)
*Tebipenem pivoxil hydrobromide*	*20/CMA 63*	*HPLC/MS*	*2.73 – 2.85*	*3.4 – 3.74*	(74)
*Ceftazidime*	*20/CMA 63*	*CE/UV*	*5*	*23.4*	(75)
*Ceftazidime*	*20/CMA 63*	*CE/C4D*	*16 – 18*	*175*	(60)
*Amoxicilline*	*20/CMA 63*	*CE/C4D*	*8*	*90*	(60)

*value estimated from the graph

** parameter of tissue penetration

ATBs, antibiotics; kDa, kilodaltons; MD, microdialysate; Cmax, maximum ATB concentrations; CMA, CMA Microdialysis, http://microdialysis.com; HPLC, high performance liquid chromatography; UV, ultraviolet-visible detection; GC, gas chromatography; MS, mass spectrometry.

### 5.1 Our experiences with microdialys in diabetic foot

**Figure 4:**
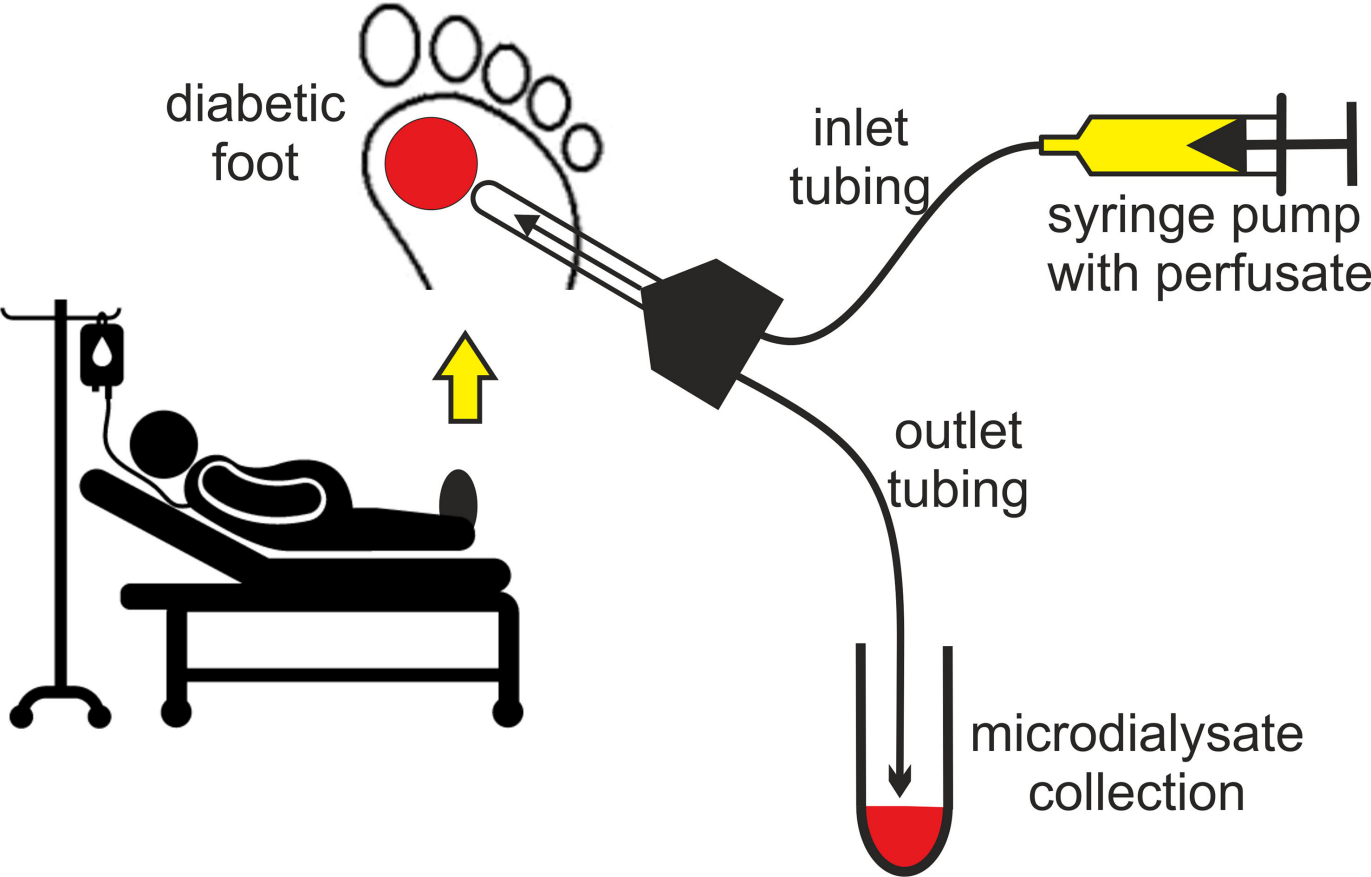
Assessment of tissue ATB concentrations using microdialysis in DF patients treated with intravenous ATBs.

Our working group has recently begun a study (DFIATIM; EudraCT No. 2019-001997-27) aimed at assessing the tissue penetration and bactericidal effects of commonly used beta-lactam and cephalosporin ATBs administered at standard doses and at different dosage regimens (bolus vs. continuous) in the context of different arterial and microcirculation status. In it, we introduce a diagnostic method for monitoring therapeutic levels of amoxicillin *(AMX)* and ceftazidime *(CTZ)* in blood plasma and MD from peripheral soft tissues of the lower limbs in patients with DF. To detect subcutaneous ATB concentrations, a microdialysis linear probe (63 Microdialysis Catheter; M Dialysis AB, Stockholm, Sweden), composed of a polyarylethersulphone (PAES) membrane with a 20 kD cut-off, was inserted close to the diabetic ulceration (**Figure 4**). The probe was then connected to a linear infusion pump filled with the acceptor solution (Ringer’s solution, see above*).*

CE-C4D method with application of large volume sample stacking was developed for sensitive determination of AMX and CTZ (60). The analysis is performed using 20 μL of serum or 15 μL of microdialysate to which is added a triplicate amount of acetonitrile. Acetonitrile provides effective deproninization of body fluids as well as suppresses their conductivity caused by the presence of NaCl. The acetonitrile-treated sample is injected into the capillary in large volumes, the length of the injected zone in this case is 5.4% of the total capillary length, but can be increased if necessary (76). Subsequently, the sample input vial is replaced with a BGE vial and the high voltage is switched on with simultaneous application of back pressure to the output vial. ATB cations migrate out of the sample zone and concentrate behind the Na^+^ zone as leading ions by transient isotachophoresis mode, and after the transfer to the BGE, ATBs separate from Na^+^ and migrate independently in the zone electrophoresis mode (77). The application of backpressure is important to push the undesirable sample matrix out of the capillary into the input vial to avoid interrupting the separation. LOQs were reduced to 148 ng/mL for AMX and 339 ng/mL for CTZ using the described method, which is sufficient to monitor therapeutic levels of ATBs in MD and serum. **Figure 3** demonstrates the pharmacokinetics of both AMX and CTZ in serum and MD of foot samples collected at 5 to 30 min intervals for 6 h after a single i.v. application of an ATB. The determined serum levels of ATBs were verified by an independent HPLC-UV method (60, 75).

This currently running study aims to find out which ATB regimens of time-dependent ATBs are more effective in DFI therapy. It is evaluated according to the maximal ATB concentrations reached during ATB application (C_max_), inhibitory coefficient (C_max_/Minimal inhibition concentration - MIC), the efficacy time (Time - T>MIC) (68), and the AUC/MIC ration (AUC - area under the plasma/tissue ATB concentrations) (69). These parameters are more reliable for the assessment of bactericidal activity of selected ATBs. Microdialysis and CE methods give us a chance to obtain C_max_ and AUC of ATBs as in blood stream as in peripheral tissue.

### 5.2 Limitations of microdialysis techniques

Microdialysis is not without its limitations, as described in detail in many previous publications (78). Specifically, the molecular weight of molecules sampled is restricted by the pore size of the dialysis membrane, determined by the cut-off value. The perfusate flows along the dialysis membrane slowly and at a constant speed. Once the dialysate is collected in microvials, it is then analysed biochemically in conjunction with enzymatic colorimetric assay. The achieved concentration of the analytes in the dialysate depends on the degree of equilibration between the interstitial fluid and the perfusate (79). The three most important factors affecting *in vivo* recovery are the length of the semi-permeable membrane, the perfusion flow rate, and the diffusion in the surrounding interstitial fluid. Recovery increases in proportion to the length of the dialysis membrane area (79). Another shortcoming is the standard cut-off of the dialysis membrane, routinely set at 20 kDa. Furthermore, the diffusion rate in the surrounding interstitial space can vary in accordance with the molecular weight of the studied analytes and the size and tortuousity of the interstitium. Recovery is thus dependent on the tissues analysed and any potential pathophysiological changes (80, 81).

Finally, linear probe insertion in regions that have been scarred due to previous surgical procedures can prove problematic. The application of microdialysis can also be painful for patients without neuropathy or haemorrhage, especially in subjects receiving anticoagulant therapy.

## 6 Summary

Microdialysis techniques are invaluable tools for determining ATB therapy efficacy in patients with DFIs. However, they have a number of limitations and therefore should be supplemented with analytical methods that are adept at determining the concentrations of substances in small-volume tissue or fluid samples. To that end, electrophoretic separation methods show promise as diagnostic tools for the detection of other target molecules and substances, which is likely to increase our understanding of the wide range of pathophysiological processes affecting especially patients with DF.

## Author contributions

VF wrote the manuscript, JP contributed to the microdialysis part and reviewed the mansucript, PT and BS contributed to the electrophoresis informations and reviewed the mansucript, RJ, JH, VW, MD reviewed the manuscript. All authors contributed to the article and approved the submitted version.
